# Case report: A novel intronic *JMJD6* likely pathogenic variant (c.941+75G > T) associated with congenital eyelid coloboma in one of the identical twin sisters

**DOI:** 10.3389/fgene.2025.1536000

**Published:** 2025-02-17

**Authors:** Xin Li, Yuqi Zhang, Gang Chai, Weijie Su, Yan Zhang

**Affiliations:** Department of Plastic and Reconstructive Surgery, Shanghai Ninth People’s Hospital Affiliated to Shanghai Jiao Tong University School of Medicine, Shanghai, China

**Keywords:** congenital eyelid coloboma, karyotype analysis, whole-exome sequencing, *JMJD6* gene, “Tenzel” flap, case report

## Abstract

**Background:**

Congenital eyelid coloboma (CEC) is a rare genetic disease, manifesting as a congenital partial or total defect of the eyelid. In this study, we report a pedigree with CEC caused by a novel pathogenic variant in *JMJD6*.

**Case report:**

The proband was a 3-year-old girl who presented with a congenital coloboma of the left upper eyelid, accompanied by hypoplasia of the ipsilateral eyebrow. Karyotype analysis was normal. Whole-exome sequencing (WES) identified a novel pathogenic variant in *JMJD6* (c.941+75G > T), which was classified as a likely pathogenic (LP) and *de novo* variant. To date, this variant has not been reported.

**Conclusion:**

Our study found a novel pathogenic variant in *JMJD6* (c.941+75G > T), which broadens the CEC phenotype spectrum and *JMJD6* gene variant spectrum, providing a basis for clinical diagnosis, genetic counseling, and treatment.

## Introduction

Congenital eyelid coloboma (CEC) is a rare congenital malformation first reported by the French physician Jacques Guillemeau in 1585. The term “coloboma” is derived from the Greek word Κολόβωμα, meaning a defect or hole in the tissue (Donaldson, 2012). CEC manifests as a congenital partial or total defect of the eyelid, leading to corneal exposure and potentially associated with eyebrow defects. Symptoms may include ocular irritation, impaired visual acuity, and even affect psychological wellbeing (Smith et al., 2015; Ng et al., 2024). It occurs more frequently in the upper eyelid than in the lower eyelid and can be either unilateral or bilateral (Smith et al., 2015). CEC is common in certain craniofacial syndromes, such as Goldenhar syndrome (Rooijers et al., 2020; Singh et al., 2020), Treacher Collins syndrome (Ng et al., 2024), and CHARGE syndrome (Lodhi et al., 2010). The etiology of CEC is unclear, involving both genetic and environmental factors, such as exposure to certain harmful substances early in pregnancy. Previous studies have suggested that congenital eyelid defects may be associated with variants in genes such as *ABCB6* (Wang et al., 2012), *FZD5* (Liu et al., 2016), *PAX6* (Azuma et al., 2003), and *SALL2* (Kelberman et al., 2014).

In this study, we report the case of a patient with congenital eyelid coloboma caused by a *JMJD6* pathogenic variant in one individual from a monozygotic twin pregnancy. Our findings expand the variant spectrum of *JMJD6* and provide novel insights into the molecular and clinical analysis of complex inherited eye disease phenotypes.

## Case presentation

**FIGURE 1:**
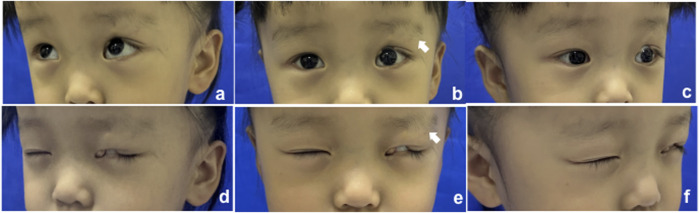
Clinical manifestation of the proband; the white arrow indicates the defect of the eyebrow. **(A–C)** Open eyes; **(D–F)** closed eyes.

**FIGURE 2:**
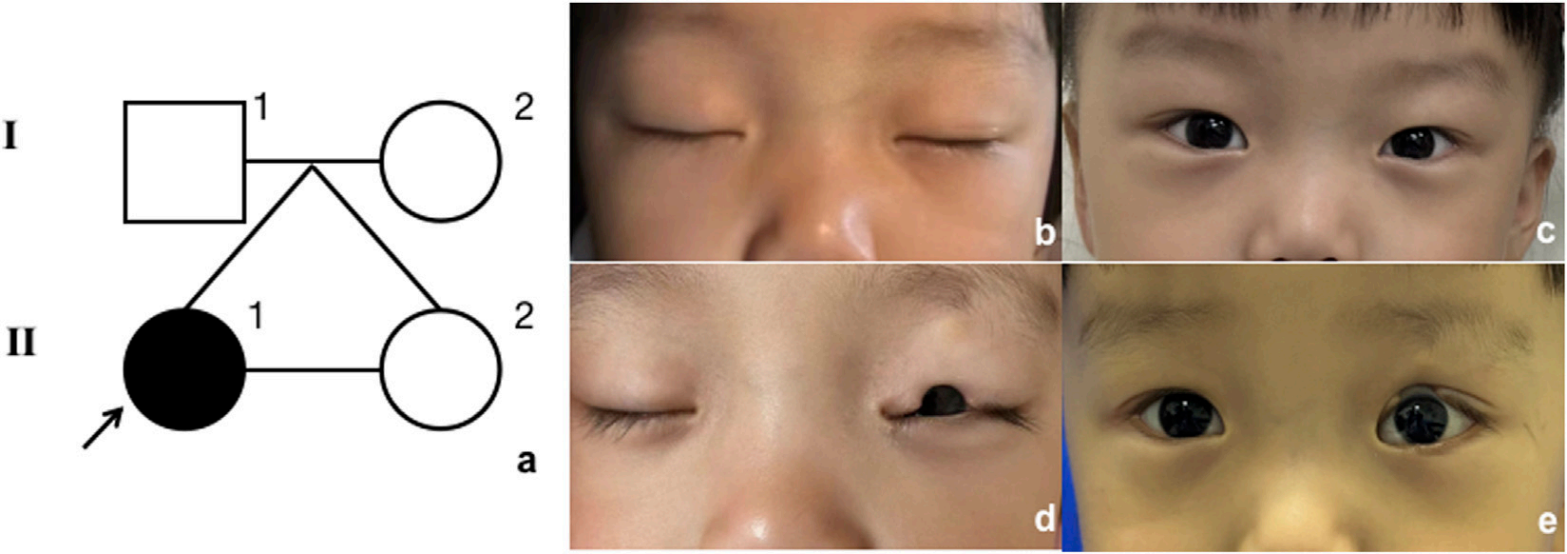
Model of inheritance. **(A)** Pedigree diagram. Black arrow indicates the proband. **(B, C)** Proband’s twin sister, closed and open eyes; **(D, E)** proband, closed and open eyes.

The proband (Figure 1, II-1) was a 3-year-old girl who presented to the Department of Plastic and Reconstructive Surgery at Shanghai Ninth People’s Hospital, Shanghai Jiao Tong University School of Medicine, with the chief complaint of a congenital coloboma of the left upper eyelid, which had been present since birth. According to the patient’s father, she was born with a full-thickness partial coloboma of the left upper eyelid measuring approximately 6 mm in length, located on the medial third of the eyelid, and was accompanied by hypoplasia of the ipsilateral eyebrow on the lateral third. Closure of the patient’s left eye revealed exposure of the left cornea (Figure 1). Slit-lamp examination revealed no obvious abnormalities in the cornea, pupil, conjunctiva, or other ocular structures, and the pupil demonstrated a sensitive response to light reflex. The presence of Bell’s phenomenon in the patient could explain the absence of corneal pathology despite significant corneal exposure. The patient did not present with any other evident congenital anomalies, including the skull, external ear, spine, and other areas. However, it is interesting that she has a monochorionic monoamniotic twin sister (II-2) from the same oosperm, who should have exactly the same genetic information as the patient, but does not exhibit any eyelid abnormalities (Figure 2). However, the patient showed no significant differences in growth, development, intelligence, or social skills compared to her monozygotic twin sister. The mother had no history of exposure to radiation or hazardous chemicals during pregnancy, and the pregnancy was uneventful without any complications. Both parents are of Han ethnicity with no history of a consanguineous marriage. There is no relevant family history of similar conditions or other congenital anomalies among the patient’s parents and other relatives.

Karyotype analysis confirmed that the patient’s karyotype is 46, XX, indicating a normal female chromosomal composition. The analysis was conducted using G-banding after cell culture. No abnormalities were observed in chromosome number (46 chromosomes) or structure (Supplementary Figure S1).

**FIGURE 3:**
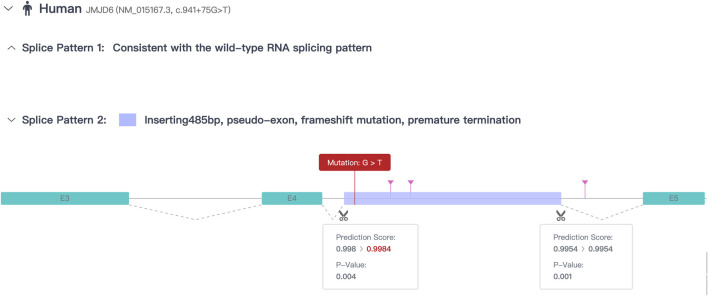
Prediction results of RDDC RNA splicer algorithms.

In addition to the clinical examinations and karyotype analysis, analysis of variants via whole-exome sequencing (WES) (Zhu et al., 2015) using peripheral blood was performed for II-1, II-2, I-1, and I-2, which was conducted by Beijing Genomics Institution Co., Ltd. (BGI, China) (Supplementary Table S1). The reference genome GRCh38 was used for sequence alignment; on average, 86,142,132 clean reads were obtained per sample, and Burrows–Wheeler Aligner (BWA) (Rajan-Babu et al., 2021) was used to align the clean reads of each sample to the human reference genome sequence. The average sequencing depth was × 104.0. An average of 122,142 single-nucleotide polymorphisms (SNPs) and 23,173 insertions and deletions (InDels) were found in all samples. First, we focused on genes with variants present in the patient but absent in the parents and the twin sibling. We then excluded genes classified as benign or with low pathogenic risk according to the American College of Medical Genetics and Genomics (ACMG) guidelines (Ghosh et al., 2017). Subsequently, genes inconsistent with the patient’s clinical manifestations were filtered out using databases such as gnomAD, ExAC, dbSNP, GeneCards, and the 1000 Genomes Project (Coppieters et al., 2016). We found the most major variant associated with the clinical features located at the heterozygous variant of *JMJD6* (NM_015167.3: c.941+75G > T), which was a *de novo* variant. The *JMJD6* variant was not recorded in the aforementioned databases. According to the ACMG guidelines (Coppieters et al., 2016), the variant is classified as “likely pathogenic” based on the presence of strong evidence (PS2: *de novo* occurrence), moderate evidence (PM2: rarity in population databases), and supporting evidence (PP1: co-segregation with the disease in the family). However, the variant was located in the intron. According to the prediction results from the Rare Disease Data Center (RDDC) RNA splicer algorithms (https://rddc.tsinghua-gd.org/) (Zhang et al., 2023), this alteration in the sequence may lead to the formation of a pseudo-exon. This results in a 485-bp insertion, causing a frameshift variant and premature termination (Figure 3). Consequently, it induces alternative splicing, affects protein coding, and leads to a splicing-related disease (Tao et al., 2024).

**FIGURE 4:**
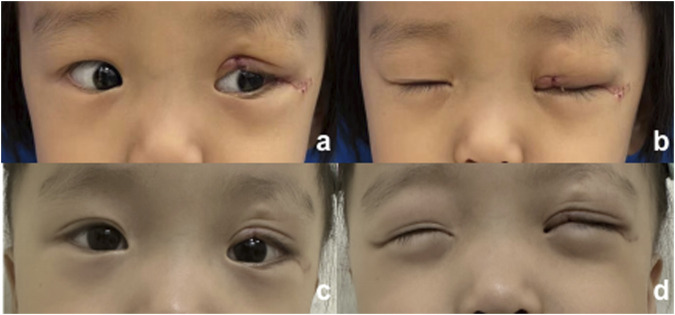
Postoperative photograph. **(A, B)** 1 week after surgery; **(C, D)** 2 months after surgery.

Due to the patient’s full-thickness eyelid defect, with the defect involving one-third to one-half of the eyelid’s horizontal length and surrounding skin in good condition, the “Tenzel” myocutaneous advancement flap (Kim et al., 2008) was selected to restore the anatomical continuity and functionality of the eyelid while maximizing the preservation of the natural appearance of the periocular area. An inverse semicircular incision was made at the lateral canthus, dissecting in a submuscular plane up to the orbital rim. Skin and orbicularis oculi flaps were created, and the superior branch of the lateral canthal ligament was identified and severed. The orbital septum was released, and the conjunctiva of the fornix was dissected and advanced anteriorly to serve as a lining for the rotational flap. Successive sutures were placed on the lower edge of the flap, which was then rotated medially. The eyelid was pulled medially to align the defect edges, and the tarsal margins were reattached with a 7-0 suture. The orbicularis oculi muscle was closed with a 5-0 suture, and the skin was sutured with 7-0 sutures. This resulted in a small dog ear inferiorly, which was excised. The flap was sutured to the periosteum at the overlap of the orbital rim with 4-0 polypropylene sutures and secured to the inferior branch of the lateral canthal ligament. The canthotomy incision was then closed with a 7-0 suture. The postoperative recovery at 1 week and 2 months is shown in Figure 4, and the patient’s guardian expressed satisfaction with the outcome, and no adverse events occurred during the treatment.

## Discussion

The gene *JMJD6*, whose full name is Jumonji domain-containing protein 6, arginine demethylase and lysine hydroxylase, is also known as the phosphatidylserine receptor (*PTDSR*). It is located at 17q25.1 and encodes a nuclear protein with a JmjC domain, which is predicted to play an important role in gene regulation through its demethylation or hydroxylation activity on histone and transcription factors (Chang et al., 2007). Bose et al. revealed that the ablation of *JMJD6* function in mice leads to delayed terminal differentiation in multiple organs, including the eyes. This disruption in eye development can result in severe abnormalities, ranging from retinal defects to unilateral or bilateral anophthalmia (Böse et al., 2004). Another study also found that *JMJD6* plays an important role in the eye development of *Xenopus* through GSK3β RNA splicing and Wnt/β-catenin signaling (Shin et al., 2019).

In our study, we identified a novel intronic variant in *JMJD6* (c.941+75G > T) through WES and associated it with CEC. This finding highlights a potential link between *JMJD6* and eyelid development. Although previous research has emphasized its role in broader ocular development, our report adds a new possible dimension by associating it with CEC, expanding the phenotypic spectrum of this gene. We acknowledge that the association between the *JMJD6* variant and eyelid coloboma is currently limited by the lack of functional validation. The identified splicing variant (c.941+75G > T) requires further experimental analysis, including RNA-level evaluation to assess its stability, processing, and translation impacts, as well as protein-level studies to confirm its pathogenicity. Functional tests are essential to elucidate the variant’s mechanistic role in eyelid development.

Interestingly, the proband’s monozygotic twin sister did not exhibit eyelid coloboma despite theoretically having identical genetic material. This suggests that the younger sister’s embryo might have acquired *de novo* variants or that environmental factors during embryogenesis influenced gene expression. The proband presented with isolated eyelid coloboma without systemic developmental or intellectual impairments, further emphasizing the significance of this specific variant.

This study reports the first case of a *JMJD6* intronic pathogenic variant (c.941+75G > T) linked to congenital eyelid coloboma, expanding the known phenotypic spectrum of *JMJD6* variants and their role in eyelid morphogenesis. Despite limitations, our findings highlight the gene’s importance in ocular development and provide insights for diagnosis and prognosis. Future research should validate the variant’s function and support diagnostic guidelines, furthering the understanding of *JMJD6*-related congenital eyelid abnormalities.

## Data Availability

The datasets presented in this study can be found in online repositories. The names of the repository/repositories and accession number(s) can be found in the article/Supplementary Material.
