# Supplementary material for: Case report: A novel intronic JMJD6 likely pathogenic variant (c.941+75G > T) associated with congenital eyelid coloboma in one of the identical twin sisters
Source: Front Genet. 2025 Feb 17;16:1536000. doi: 10.3389/fgene.2025.1536000 (PMC11872945; doi:10.3389/fgene.2025.1536000)
Supplement: Supplementary file 1 [file DataSheet1.pdf]

Supplementary Material

Supplementary Figures

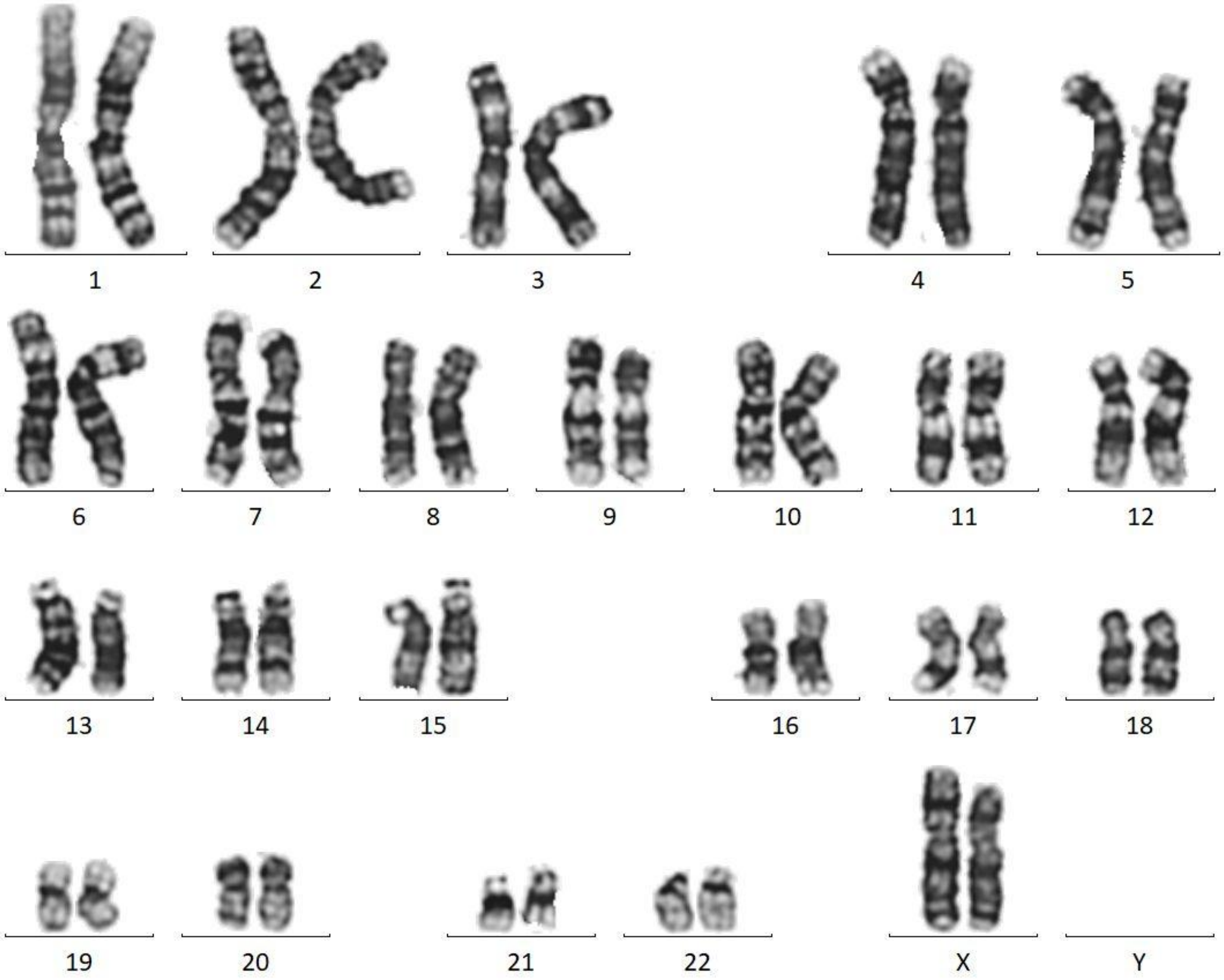

Supplementary Figure 1. The karyotype analysis of the proband.
